# ‘Best interests’ in paediatric intensive care: an empirical ethics study

**DOI:** 10.1136/archdischild-2016-312076

**Published:** 2017-04-13

**Authors:** Giles Birchley, Rachael Gooberman-Hill, Zuzana Deans, James Fraser, Richard Huxtable

**Affiliations:** 1 Centre for Ethics in Medicine, University of Bristol, Bristol, UK; 2 Musculoskeletal Research Unit, University of Bristol, Bristol, UK; 3 Paediatric Intensive Care Unit, Bristol Royal Hospital for Children, Bristol, UK

**Keywords:** Paediatric Intensive Care Units, Decision Making, Ethics, End-of-Life-Care, Law

## Abstract

**Objective:**

In English paediatric practice, English law requires that parents and clinicians agree the ‘best interests’ of children and, if this is not possible, that the courts decide. Court intervention is rare and the concept of best interests is ambiguous. We report qualitative research exploring how the best interests standard operates in practice, particularly with decisions related to planned non-treatment. We discuss results in the light of accounts of best interests in the medical ethics literature.

**Design:**

We conducted 39 qualitative interviews, exploring decision making in the paediatric intensive care unit, with doctors, nurses, clinical ethics committee members and parents whose children had a range of health outcomes. Interviews were audio-recorded and analysed thematically.

**Results:**

Parents and clinicians indicated differences in their approaches to deciding the child’s best interests. These were reconciled when parents responded positively to clinicians’ efforts to help parents agree with the clinicians’ view of the child’s best interests. Notably, protracted disagreements about a child’s best interests in non-treatment decisions were resolved when parents’ views were affected by witnessing their child’s physical deterioration. Negotiation was the norm and clinicians believed avoiding the courts was desirable.

**Conclusions:**

Sensitivity to the long-term interests of parents of children with life-limiting conditions is defensible but must be exercised proportionately. Current approaches emphasise negotiation but offer few alternatives when decisions are at an impasse. In such situations, the instrumental role played by a child’s deterioration and avoidance of the courts risks giving insufficient weight to the child’s interests. New approaches to decision making are needed.

**What is already known on this topic?**

- While English law says best interests encompass more than medical interests, best interests are poorly defined.
- English law states that when parents and clinicians cannot agree to the best interests of a child, the courts should be consulted.
- Little is known about the processes by which best interests decisions are made in Paediatric Intensive Care Units.

**What this study adds?**

- Deciding best interests relies on a process where clinicians encourage parents that the medical view of their child’s best interests is correct.
- Where opinion is at impasse, clinicians avoid the courts, and deterioration of the child can be instrumental in helping parents understand the child’s best interests.
- The instrumental role played by a child’s deterioration and avoiding the courts risks losing sight of the burdens prolonged intensive care place upon the child.

## Introduction

Between 2004 and 2013, admissions of children to paediatric intensive care units (PICUs) in the UK rose by 15%. This increase was in part due to advances in life-sustaining technology and increasing numbers of children surviving with life-limiting conditions.^1 2^ Most deaths in the PICU follow decisions to withdraw or limit treatment.^3^ The majority of PICU patients are ventilated and three-quarters are under 5 years old.^2^ Such patients are unable to participate in decision making.

‘Best interests’ is the international ethico-legal standard by which decisions are made about children.^4 5^ Although ‘best interests’ are conceptually ambiguous, English law states that the child’s best interests are the paramount consideration in any decision,^6^ and holds that best interests go beyond medical interests to encompass ‘medical, emotional and all other welfare issues.’^7^ Legal and professional guidance states parents and clinicians should share ‘best interests’ decisions^8^ and in non-emergency situations the courts should be involved if agreement cannot be reached.^9^ Thus, the current situation resembles shared decision making, a defining (although still ambiguous) feature of contemporary doctor–patient relationships.^10^

The ambiguities of the decision-making process create the potential for conflict.^11^ Although legal records indicate lengthy and sometimes acrimonious negotiation between parents and clinicians,^12^ recourse to the courts appears rare^8^ and relatively little is known about what happens in practice. This study explores how decisions are made, with a particular focus on decisions about (non-)treatment. Our objective is to critically describe the way in which the best interests standard operates in PICU, with reference to theories of ‘best interests’ from the medical ethics literature.

## Methods

The study is a qualitative interview study embedded in a larger ‘empirical ethics’ study. Empirical ethics is a methodology increasingly used in bioethics to ensure that practical experiences inform theoretical analysis.^13^ Empirical ethics methodologies arise from concern that philosophical medical ethics has not adequately considered the experiences and social contexts of patients or doctors. Empirical ethics methodologies vary but broadly comprise processes that critically synthesise theoretical and empirical sources.^14^ Our study used a method known as ‘reflective equilibrium’^15^ to synthesise a literature review and results of a qualitative study. In this article, we present the qualitative study that was part of this process and interpret the qualitative findings in relation to relevant medical ethics literature.

Participants in the qualitative study comprised key decision makers at three English PICUs. Following practice guidelines,^16 17^ we defined key decision makers as parents, doctors, nurses and members of clinical ethics committees.

Parents were eligible to participate if their child had been admitted to PICU within the past 2 years (to minimise duress), was <4 years old (to ensure the child expressed no antecedent wishes) and was critically ill with length of admission >4 days (to ensure adequate experience of PICU). Parents were sampled purposively to reflect potential health outcomes of a PICU admission. Parents were approached by letter from their treating PICU and were asked to send a reply slip to the study team if they wished to discuss participation. Doctors, nurses and members of clinical ethics committees were approached if they had experience of PICU decisions.

A total of 234 potential participants were approached in writing and 49 people replied. Interviews and analysis took place iteratively until thematic saturation was achieved,^18^ which occurred at 39 interviews and recruitment was ceased. The final sample comprised 14 parents, 10 doctors, 8 nurses and 7 members of clinical ethics committees (table 1).

**Table 1:** Recruitment

**Group**	**Study site**	**Approaches**	**Responses**	**Consents**	**Interviews**	**Total**
**Parents**	Site 1	71	11	8	8	14
	Site 2	40	3	3	3	
	Site 3	20	3	3	3	
**Doctors**	Site 1	10	6	4	4	10
	Site 2	5	3	3	3	
	Site 3	4	3	3	3	
**Nurses**	Site 1	20	8	6	6	8
	Site 2	10	3	2	2	
**Clinical ethics committee**	Site 1	10	5	5	5	7
	Site 2	(44)*	4	2	2	
					Total	39

*At site 2, the committee chair circulated the details of the study to members and invited them to respond directly to the chief investigator.

Parents were given the option to be interviewed alone or with a partner (table 2). Interviews explored participants’ experiences of decision making. Interview topics, including scope of parental discretion and acceptable quality of life, were developed from existing literature. Interviews lasted 40–180 min and consent was provided immediately before each interview.

**Table 2:** Characteristics of parents

ID*	**Participant(s) in interview**	**Age of child**	**Admission type**	**Length of admission^†^**	**LLC^‡^**	**Outcome of admission^§^**
**P40**	Both parents	<1 month	Emergency	>1 month^¶^	Yes	Ongoing ill health
**P41**	Both parents	<1 month	Emergency	>1 month^¶^	Yes	Death
**P42**	Mother**	1–12 months	Emergency	<2 weeks	Yes	Death
**P45**	Both parents	1–4 years	Emergency	2–4 weeks	No	Ongoing ill health
**P55**	Mother	1–4 years	Emergency	2–4 weeks^**^†^^†^**^	No	Recovery
**P56**	Both parents	1–12 months	Elective	<2 weeks	No	Recovery
**P58**	Mother	1–4 years	Emergency	<2 weeks	No	Ongoing ill health
**P59**	Both parents	1–4 years	Emergency	<2 weeks	Yes	Ongoing ill health
**P60**	Mother	<1 month	Emergency	2–4 weeks	No	Recovery
**P61**	Mother	1–4 years	Emergency	<2 weeks	No	Ongoing ill health
**P62**	Mother	1–4 years	Elective	<2 weeks	No	Recovery
**P63**	Mother**	1–4 years	Elective	<2 weeks	No	Recovery
**P64**	Mother	<1 month	Emergency	<2 weeks	No	Recovery
**P65**	Both parents	1–12 months	Emergency	<2 weeks	No	Recovery

*Participant identifier.

†Period of time given to aid anonymity.

‡Life-limiting condition, as categorised by Hain and Devins.^40^

§Recovery is where children leave paediatric intensive care unit (PICU) with an improvement in their preadmission baseline health; ongoing ill health is where the children leave PICU with a deficit to their baseline health.

¶Includes time on ward due to multiple readmissions to PICU during hospital stay.

**A supportive other was also present but did not participate in the interview.

††Total length of stay on ward plus PICU as times were unclear.

Interviews were transcribed, anonymised and analysed thematically.^19^ The first author assigned codes to the data, with codes derived inductively. Data were then grouped into broader themes. Ten per cent of transcripts were second coded independently by members of the study team, and this process was used to refine codes and theme development.^20^

### Ethics

The study was funded by the Wellcome Trust (grant number WT097725MF) and received Research Ethics approval from the Southwest NHS Research Ethics Committee (reference 12/SW/0210). All participants gave written, informed consent, including to audio-recording and publication of anonymised quotations.

## Results

Analysis of interviews with study participants gave rise to three central themes that covered the ideal principles and practical processes of decision making and the role of the courts:

### How should decisions be made?

Most participants thought that decisions should be shared between clinicians and parents but did not necessarily agree how this should occur. Parents were unanimous that the child alone should be the focus of the decision but felt that families should be allowed to make independent decisions where the life of their child was at stake. Doctors considered collaboration with families to be standard practice but voiced concerns about the impact on parents of sharing life or death decisions. Some clinicians explicitly conflated child and parent interests or emphasised the interests of the parents where prognosis was poor (Box 1).

Box 1 **How should decisons be made?**

Parents:

- P58 (Mother): I think that it should be more to do with the child’s wellbeing and quality of life than the parents’ wellbeing and quality of life that is taken into consideration. Um it should really be centred on them rather than the parents.
- P64 (Mother): I think if you’re the parent, I don’t know, you’ve got to see what’s right for the child. Not what’s right for you. Not what’s right for the hospital. Not what’s right for the doctors. You’ve got to see what’s right for the child.
- P60 (Mother): how could you live with yourself if you hadn’t looked yourself at the options. Not because you don’t trust the medical profession, or that they haven’t got the best interest of your child, but because you just need to have looked and exhausted every avenue. It’s the parents’ conscience that it has got to live on for the rest of their lives. … If you regretted or felt that you had failed your child at any point, that’s too much to live with, isn’t it?

Clinicians:

- D34 (Doctor): Oh it’s a joint thing. … I think there is a real balance between the doctor being the kind of god who decides, you know, which is very unpopular at the moment, but on the other hand neither can you put all that responsibility onto the parents. Because, yeah, how could [parents] live with [themselves] having taken that decision?
- N37 (Nurse): I think at times you have to … remember almost that when it says best interests of the child it should more than likely say at times the best interests of the child and their family.
- D46 (Doctor): the parents have to go home and live with these decisions for the rest of their lives. So if there is some way that we can make the decisions seem reasonable and acceptable and the right thing to them, even if it involves more time, which it usually does, then that might be a reasonable thing to do.

### What happens in practice?

#### Parents’ views

Parents’ views about what ought to occur did not always equate to what happens in practice. While parents accepted clinical expertise, some acknowledged that deferring to doctors’ advice compromised their stated desire for independence. In order to accept medical opinion, parents had to trust the doctor and relinquish their own authority over their child. Some parents said that a limited range of decision making for critical decisions was left in their hands. Other parents felt they had no real choices in decisions but were given the impression that they had choices, which they found reassuring.

#### Clinicians’ views

Clinicians described distinctive strategies they used in discussions with parents. These encompassed a process of advocacy to advance the clinical view of best interests. Initially they conveyed technical information to allow parents to understand the medical perspective. If a child’s prognosis was poor and parents did not share the clinicians’ view of the child’s ‘best interests,’ clinicians reframed their description of the medical plan in terms they thought would be more acceptable to the parents. If not effective, starker explanations were offered to parents.

Clinicians felt the success of advocacy required sensitivity to parents’ states of mind. For instance, they explained that if there was no hope of improvement, then they would allow time to pass so parental feelings could adjust. In this context, clinicians described the vital role played by the physical appearance of a child in moving towards a decision, as appearance enabled parents to witness their child’s deterioration and validated the clinical view of ‘best interests.’ While other signs such as frequent admissions to PICU were sometimes cited as corroborators, clinicians thought the physical appearance of the child was most important to parents when considering the child’s best interests. For instance, when the child’s best interests lay in non-treatment, a child’s relatively ‘abnormal’ appearance at admission could help parents understand the prognosis (and thus agree to non-treatment) more easily than when a child appeared to be either visually or behaviourally more ‘normal’ (Box 2).

Box 2 **What happens in practice?**

Parents:

- P41 (Mother): I don’t think it is an equal relationship. I think that you do need guidance from a doctor. I don’t mean, when I say guidance, I don’t mean guiding [parents] towards a particular answer. I mean guiding [parents] towards being in a position to be able to make a decision.
- P59 (Mother): I think doctors should – should just – well they’ve gotta give their opinion and maybe say, “I think it’s for the best, but it’s up to you,” sort of thing. ‘Cos then if a doctor says to you, “I think it’s for the best,” you’re – nine times out of ten you’re gonna think, “Well if the doctor’s thinking that, and he’s a, you know, a doctor, maybe we should listen to him and sort of do the right thing.
- P61 (Mother): But I don’t really feel that there was a decision for me to make. This was just so beyond for me to make a decision on anything. It was just like, "Well yes, you do what you have to do."
- P65 (Father): when it comes to somebody else making a decision about your child, no matter how minor it is, most parents would want to be involved with that decision. … I just find it hard to let someone else make a decision, especially when I don’t know if it’s right or not. If I knew it was right, then I’d be more than happy for anyone else to make the decision. I dare say a lot of parents feel like that. So if there’s a massive amount of stuff like we had. I was always, whatever they suggested that they needed to do, I was happy for them to do that. Because I had the confidence that they knew what they were doing.
- P56 (Father): [the consultant] does a very good job of making it seem like it’s a choice, even if it isn’t achoice. Um but that’s the important thing for me: it seems like a choice. … You’re never gonna say,“No I don’t wanna do this, because I don’t want my baby to get better” … So there is no choice, but the illusion is there that you could if you wanted to.
- P42 (Mother): again it comes back to that control. I had con—even though I probably didn’t—I had control over the whole thing. Um medically, behind the scenes I probably didn’t, [but] even talking to you now, I felt that we had made that decision and we—actually there was no other decision to make.

Clinicians:

- N29 (Nurse): you can be brutally honest with the parents, and support them.
- D44 (Doctor): something about the parents and the way they were responding … told me that it just wasn’t the right time, that we needed yet another episode of intensive care.
- N48 (Nurse): you will probably find us introducing the elements and snippets of information so that [parents] get used to it; not giving it all at once. And so you find that they will come to their own decision.
- D27 (Doctor): when [parents] see them, you know, changing colour or getting puffy or not looking like their child … they have either had time to rationalise it in their mind or speak to their partner or just come to terms with it.
- D34 (Doctor): And the poor little thing didn’t look promising [describes abnormal physical appearance]. Which I think had been good for the family, because I think they could see very early on that this wasn’t a runner.
- N29 (Nurse): they [parents] might think they’re doing really well today because the nurse on the night shift has got them dressed and put them in a Babygro, so they look – they look like their baby. Whereas in fact all the other intensive care stuff is exactly the same.

### Avoiding the courts

Few participants had direct experience of the courts’ involvement in resolving disputes about best interests. Many parents questioned whether it was appropriate for judges to make decisions about their children. Clinicians believed judicial decisions were inconsistent and felt approaching the courts was arduous, divisive and lengthy. Some clinicians believed the legal framework pressured them to agree to demands from families that they described as ‘unreasonable.’ Others felt that resolving conflicts about a child’s best interests without using the courts was a measure of personal or institutional success (Box 3).

Box 3 **Role of the courts**

Parents:

- P45 (Mother): how do you relay all the facts to that judge, and how do they understand it? Because,yes, there is emotive stuff, and there’s ethics, and there’s all sorts of other things in there, but at the end of the day there are a lot of medical things in there as well which, if the judge isn’t au fait um with those sort of factors then it’s quite—I don’t know—I’d find it a little bit strange that you’d want somebody from a non-medical background sort of making that opinion.
- P59 (Father): [discussing judges] I don’t know how the law can allow anyone to take that decision out of your hands: it’s your child. I think it’s wrong.

Clinicians:

- N38 (Nurse): I do think that maybe sometimes we end up going more favouring towards the parents and what they think is right probably, because we want to avoid that going to court.
- D34 (Doctor): I may have my expertise and opinions as to what to do, but basically somebody with no knowledge, no requirement to be reasonable in any shape or form, can come and demand that I do something … On the whole, that’s not what you’re faced with. But you can be. You can just get an angry, unreasonable family who “know their rights,” you know. And the cards are all stacked in theirhands. It’s very difficult to kind of not treat in that sort of situation.
- D44 (Doctor): But I have never been in a situation where it’s been that far that I’ve had to use a judge. … Some of my colleagues have had to do that, yes. I haven’t. I um please myself in thinking, because I’ve had many cases, in thinking that it’s maybe because I’ve managed to communicate with parents in such a way that they trust me. But that’s just, you know, that’s just pleasing myself in thinking that; I don’t know if it’s true.

For these participants, shared decision making about the best interests of a child was a process where clinicians encourage parents to adopt their viewpoint. Persuasion was acceptable to most parents in this study. If the persuasive process stalled over disagreements about whether non-treatment was in the child’s best interests, clinicians interviewed in this study felt they had few options. Parents who firmly refused consent were thought to exert considerable leverage in decisions to continue intensive care even when clinicians regarded that care as too burdensome for the child. If the process reached a ‘stalemate’ there was reluctance to approach the courts to resolve disagreement. Such views arose from doubts regarding the efficacy of the legal processes and judgements.

## Discussion

Many ethicists accept parental authority as a mechanism to decide the best interests of children although it is usual to impose some limits on this authority. Moreover, some suggest it is acceptable to allow families to take into account considerations of family interests other than those of the child,^21 22^ questioning the validity of best interests as a decision-making standard in this respect. They suggest other approaches including the ‘harm standard’, which allows parental discretion in all cases save those where extreme and irremediable harm would ensue.^23^ Others observe that differences between families’ abilities to benefit children drive inequality,^24 25^ and suggest that children’s vulnerability justifies additional decision-making safeguards.^26^ Although the legal position outwardly prioritises the interests of the child,^6^ medical interests alone do not determine best interests, resulting in judges sometimes focusing on burdens to parents as well as the child.^12^

Our empirical research suggests that, although all parties were concerned with the child’s welfare, clinicians were sympathetic to perspectives that emphasise family interests. In contrast, parents suggested their focus was solely on their child’s welfare, and aspired to make these decisions themselves, while recognising clinical prerogatives in this area. While parents’ responses may result from their failure to identify their own inherent interests, their perspective may be a barrier to their acceptance of approaches that emphasise parental welfare. More, if this is a case where clinicians know better than parents, clinical expertise in deciding best interests lacks overt recognition in ethical discussion of children’s best interests. Similarly, our finding that clinicians steered reluctant parents towards clinical plans may be unsurprising for practitioners but, again, is not necessarily reflected in current literature.

Increasingly broad understandings of parental authority^27–29^ risk characterising parent–doctor interactions as a conflict between autonomy and paternalism. Certainly the common language of ‘informed consent’ can draw inappropriate parallels between the authority of a *patient* and the authority of a *parent*. The risks of this approach in decisions about non-treatment are evident in our findings; allowing the parent to personally experience the deterioration of their child where they doubt that the best interests of their child lie in non-treatment. Such a strategy appears commonplace. For instance, a recent study of withdrawal of treatment on neonatal intensive care unit (NICU) noted that physicians gave resistant parents ‘more time to be with their child and to witness the ongoing deterioration despite full [intensive care] support.’^30^ Since we also found clinicians were reluctant to approach the courts when agreement could not be reached, this strategy places an emphasis on protracted discussion that risks considerable suffering to the child. Indeed, it may also harm the interests of the parents, since the long-term benefits to parents who perceive that they were involved in end-of-life decisions appear jeopardised when parents believe their child has suffered.^31^

English law states that the best interests of a child are ‘the paramount concern,’^6^ which is often seen to mean that the child’s interests alone will determine the outcome of any decision.^32^ Common law shows more flexibility with the interests of parents sometimes playing an implicit part in judgements of the child’s best interests.^12^ Rather than ‘the paramount concern,’ the United Nations Convention on the Rights of the Child suggests a child’s best interests are ‘a primary consideration,’^33^ a difference that arguably extends the scope of a best interests decision to include other significant interests.^5^ It is notable that this wording is reflected in recent clinical guidelines.^17^ While the law does not explicitly acknowledge that clinicians have a duty to parents as well as the child, the serious risks of morbidity and mortality attached to parental bereavement^34^ imply an ethical duty to both child and parents exists, and clinicians’ attention to parental interests reflects this understanding. However, risks to parents must be balanced against the suffering that children experience during prolonged intensive care which may be inadequately mitigated by analgesia and sedation.^35^ Where there is disagreement about the best course of action, clinicians should consider how to equitably balance a child’s suffering with the benefits of parental involvement in decision making. Since clinicians interviewed in this study lacked confidence in the courts to resolve impasse, achieving this balance apparently requires other alternatives to the consensus approach.

Our study suggests that the decision-making environment in PICU lacks mechanisms to ensure a balance between parents’ and children’s interests is maintained. New decision-making standards may be needed. An ideal alternative standard would explicitly recognise that a variety of interests are involved and compel clinicians to include parents in decision making while providing a route to expeditious resolution of impasse should equipoise be lost between the child’s and parent’s interests. It is not clear that current alternatives described within the medical ethics literature, which are dominated by the harm standard, can fulfil these criteria. Harm is not always self-evident and this approach may struggle to clarify the limits of parental discretion.^36^ While future research should aim to identify and finesse alternative standards, we suggest the concept of parental *assent* (rather than consent) is worthy of future investigation as a way of striking the correct balance.

### Novelty and limitations

This study makes a number of important contributions to our understanding of best interests. While decision making in the NICU has been the focus of numerous empirical studies,^30 37–39^ few studies specifically consider best interests in decision making in PICU, which has its own unique challenges. The emphasis that parents and clinicians in PICU place on the interests of the family, and the attitudes of PICU decision makers to court advice have never, to our knowledge, been investigated before. Moreover, few studies give empirical evidence of the persuasive strategy used in shared decisions about the best interests of the child. While the instrumentalisation of the child in decision-making practice has been noted elsewhere, ethical critique of this is novel.

This study has a limitation since qualitative research findings are not intended to be generalisable. However, drawing the sample population from multiple sites, using purposive sampling to include a range of individuals and a variety of children’s outcomes, and achieving thematic saturation go some way in providing confidence that the qualitative findings are sound and transferable.

## Conclusion

Decisions about treatment of children are governed by ambiguous concepts of best interests and shared decision making. Our research indicated differences in the approaches of parents and clinicians to these concepts. Where best interests were disputed, clinicians indicated strategies to encourage parents to adopt the clinical view. Where disputes were protracted, the courts were avoided and the deteriorating physical appearance of the child could play a key role. Parents and children both have important interests at stake in PICU, but these may conflict. Current processes cannot ensure a balance between these conflicting interests is maintained, and new decision-making standards may be needed.
